# Supplementary material for: Differential Expression of the Apolipoprotein AI Gene in Spotnape Ponyfish (Nuchequula nuchalis) Inhabiting Different Salinity Ranges at the Top of the Estuary and in the Deep-Bay Area of Gwangyang Bay, South Korea
Source: Int J Environ Res Public Health. 2021 Oct 19;18(20):10960. doi: 10.3390/ijerph182010960 (PMC8536079; doi:10.3390/ijerph182010960)
Supplement: Supplementary file 1 [file ijerph-18-10960-s001.zip › ijerph-1366850-supplementary.pdf]

Supplementary Table S1. Species information used in phylogenetic analysis.

Species Name	Gene	GenBank Accession Number
N. nuchalis in the study	ApoAI	MZ851746
Etheostoma cragini (Gilbert, 1885)	ApoA-Ib	XP_034724041
Perca fluviatilis (Linnaeus, 1758)	ApoAI-like	XP_039643066
Morone saxatilis (Walbaum, 1792)	ApoAI-like	XP_035536036
Echeneis naucrates (Linnaeus, 1758)	ApoAI-like	XP_029373375
Seriola lalandi dorsalis (Valenciennes, 1833)	ApoA-IV	XP_023256300
Micropterus salmoides (Lacepède, 1802)	ApoAI-like	XP_038585910
Rachycentron canadum (Linnaeus, 1766)	ApoAI	ACV50420
Sander lucioperca (Linnaeus, 1758)	ApoA-Ib	XP_031139526
Toxotes jaculatrix (Pallas, 1767)	ApoA-Ib	XP_040903009
Seriola dumerili (Risso, 1810)	ApoA-IV	XP_022616173
Anoplopoma fimbria (Pallas, 1814)	ApoAI precursor	ACQ58664
Perca flavescens (Mitchill, 1814)	ApoAI-like	XP_028427328
Scophthalmus maximus (Linnaeus, 1758)	ApoAI-like	XP_035478339
Lates calcarifer (Bloch, 1790)	ApoAI-like	XP_018543131
Astatotilapia calliptera (Günther, 1894)	ApoA-IV	XP_026047886
Liparis tanakae (Gilbert & Burke, 1912)	ApoAI	TNN34591
Epinephelus coioides (Hamilton, 1822)	ApoAI	ACM48181
Epinephelus akaara (Temminck & Schlegel, 1842)	ApoAI	AGT02114
Pungitius pungitius (Linnaeus, 1758)	ApoA-Ib	XP_037317114
Larimichthys crocea (Richardson, 1846)	ApoAI	XP_010743548
Parambassis ranga (Hamilton, 1822)	ApoAI-like	XP_028275332
Channa argus (Cantor, 1842)	ApoAI	KAF3693043
Notothenia coriiceps (Richardson, 1844)	ApoAI	XP_010792180
Chelmon rostratus (Linnaeus, 1758)	ApoA-Ib	XP_041796720
Cyclopterus lumpus (Linnaeus, 1758)	ApoA-Ib	XP_034404235
Epinephelus lanceolatus (Bloch, 1790)	ApoA-Ib	XP_033487725
Gymnodraco acuticeps (Boulenger, 1902)	ApoAI-like	XP_034058693
Oryzias melastigma (McClelland, 1839)	ApoAI	AEA51138
Gasterosteus aculeatus (Linnaeus, 1758)	ApoAI-like	XP_040046834
Anabas testudineus (Bloch, 1792)	ApoAI	XP_026230177
Hippoglossus hippoglossus (Linnaeus, 1758)	ApoA-Ib	XP_034459733
Fundulus heteroclitus (Linnaeus, 1766)	ApoAI	XP_012728061
Collichthys lucidus (Richardson, 1844)	ApoAI	TKS81356
Austrofundulus limnaeus (Schultz, 1949)	ApoA-IV	XP_013881474
Xiphias gladius (Linnaeus, 1758)	ApoA-Ib	XP_039987748
Homo sapiens	ApoAI	X02162
Mus musculus	ApoAI	NM_009692
Rattus norvegicus	ApoAI	NP_036870
Macaca fascicularis	ApoAI	NP_001270674
Saimiri boliviensis	ApoAI	XP_010332821
